# Patient-specific instrumentation improved axial alignment of the femoral component, operative time and perioperative blood loss after total knee arthroplasty

**DOI:** 10.1007/s00167-018-5256-0

**Published:** 2018-10-30

**Authors:** Song Gong, Weihua Xu, Ruoyu Wang, Zijian Wang, Bo Wang, Lizhi Han, Guo Chen

**Affiliations:** 1 Department of Orthopaedics, Union Hospital, Tongji Medical College, Huazhong University of Science and Technology, Wuhan, 430022 China; 2 Department of Rehabilitation, Union Hospital, Tongji Medical College, Huazhong University of Science and Technology, Wuhan, 430022 China

**Keywords:** Total knee arthroplasty, TKA, Patient-specific instrumentation, PSI, Standard instrumentation, SI, Alignment

## Abstract

**Purpose:**

The purpose of the present study was to compare patient-specific instrumentation (PSI) with standard instrumentation (SI) in patients undergoing total knee arthroplasty (TKA). PSI is hypothesized to have advantages with respect to component alignment; number of outliers (defined as alignment > 3° from the target alignment); operative time; perioperative blood loss; and length of hospital stay. This new surgical technique is expected to exhibit superior performance.

**Methods:**

A total of 23 randomized controlled trials (RCTs) involving 2058 knees that compared the clinical outcomes of TKA between PSI and SI were included in the present analysis; these RCTs were identified via a literature search of the PubMed, Embase, and Cochrane Library databases through March 1, 2018. The outcomes of interest included coronal, sagittal and axial component alignment (presented as the angle of deviation from the transcondylar line); number of outliers; operative time; perioperative blood loss; and length of hospital stay.

**Results:**

There was a significant difference in postoperative femoral axial alignment between PSI and SI patients (95% CI − 0.71 to − 0.21, *p* = 0.0004, *I*^2^ = 48%). PSI resulted in approximately 0.4° less deviation from the transcondylar line than SI. Based on our results, PSI reduced operative time by a mean of 7 min compared with SI (95% CI − 10.95 to − 3.75, *p* < 0.0001, *I*^2^ = 78%). According to the included literature, PSI reduced perioperative blood loss by approximately 90 ml compared with SI (95% CI − 146.65 to − 20.18, *p* = 0.01, *I*^2^ = 74%). We did not find any differences between PSI and SI with respect to any other parameters.

**Conclusions:**

PSI has advantages in axial alignment of the femoral component, operative time, and perioperative blood loss relative to SI. No significant differences were found between PSI and SI with respect to alignment of the remaining components, number of outliers, or length of hospital stay.

**Level of evidence:**

Therapeutic study (systematic review and meta-analysis), Level I.

## Introduction

According to reports, the rate of component malpositioning can be 20% to 40% using standard instrumentation (SI) [7, 18], and component positioning is an essential factor that affects postoperative functional recovery, patient satisfaction, and especially long-term component survival [14, 46]. In recent years, the introduction of patient-specific instrumentation (PSI) has gradually become popular among orthopaedic surgeons and is expected to improve component alignment and positioning, postoperative functional recovery, and patient satisfaction [8, 35]. The fundamental processes are preoperative computed tomography (CT) and/or magnetic resonance imaging, computer-aided three-dimensional (3D) reconstruction, 3D printing from a disposable template, accurate intraoperative placement and osteotomy. Several meta-analyses have compared the application of PSI to that of SI for total knee arthroplasty (TKA) in recent years, but no comprehensive systematic review and meta-analysis has been published [2, 9, 17, 19, 30, 42, 45, 51–54, 60]. PSI is hypothesized to have advantages with respect to improving component alignment, shortening the surgical time and length of hospital stay, and decreasing perioperative blood loss.

## Materials and methods

A literature search was performed in the PubMed, Embase, and Cochrane Library databases following the recommendations of the Cochrane Collaboration and the Preferred Reporting Items for Systematic Reviews and Meta-Analyses statement. The Cochrane Central Register of Controlled Trials was searched using the following terms: total knee arthroplasty, TKA, total knee replacement, TKR, standard instrumentation, conventional instrumentation, patient-specific instrumentation, PSI, patient-matched, customised instrumentation, and custom cutting block. The searches were restricted to the English language. Two independent reviewers (SG and RYW) selected the articles obtained from the PubMed, Embase, and Cochrane Library databases. Disagreements between the reviewers were resolved by consulting a superior (WHX) to reach a consensus.

### Inclusion and exclusion criteria

1. Studies of TKA comparing PSI with SI in terms of at least one of the following: coronal, sagittal and axial component alignment; number of outliers; operative time; perioperative blood loss; and length of hospital stay, were included
2. Randomized controlled trials (RCTs)
3. Minimum of 40 patients in both the PSI and the SI TKA groups
4. Patients older than 18 years
5. Studies with an Improved Jadad Rating Scale score of less than 3 were excluded
6. Fracture, deformity, tumour, animal and cadaver studies were excluded
7. Studies exclusively reporting unicondylar knee component outcomes were excluded

To ensure a high-quality analysis, studies involving RCTs and a strict Improved Jadad Rating Scale score of at least 3 were included. The patients were required to be of legal age (at least 18 years old) to ensure that they had the right to sign the consent form for the surgery. Studies lacking any of the above-mentioned inclusion criteria or involving any of the above-mentioned exclusion criteria were excluded.

### Data collection and methodological quality assessment

Two reviewers (SG and ZJW) independently extracted the following data from each study: first author, country of origin, Improved Jadad Rating Scale score, number of patients, mean age, pre-imaging results, gender ratio, body mass index (BMI), PSI system, accuracy of component alignment, number of outliers, surgical time, perioperative blood loss, and length of hospital stay. Several of the initial articles contained some indicators of the means and 95% confidence intervals (CIs), which were converted to the means and standard deviations [63]. The deviation angle from the target alignment is expressed as an absolute value. The methodological quality evaluation included all studies, which were graded using the seven-point Improved Jadad Rating Scale. This widely used scale evaluates the reporting of studies based on four fundamental methodological criteria: the method of randomization, reasonable allocation concealment, adequacy of blinding, and description of withdrawals and dropouts. The quality was classified to as high (score of 4–7) or low (score of 0–3) [36, 37, 63]. The minimum score for inclusion in our study was 3, and all but one of the included studies were evaluated as high-quality. The numbers of patients in the test and control groups were extracted from each article, resulting in a total of 2058 patients. Any disagreements regarding study quality evaluation were resolved by reviewing the study in question and discussing discrepancies.

### Statistical analysis

The statistical analysis was performed using Review Manager version 5.3 (The Cochrane Collaboration, Oxford, UK). For each study, we calculated risk ratios (RRs) with 95% CIs for dichotomous data and mean differences with 95% CIs for continuous data. Where appropriate, we pooled the results of comparable groups of trials using a fixed-effect model (via the Mantel–Haenszel test) or a random-effect model (via the DerSimonian–Laird method). A random-effect model was used when significant heterogeneity was detected among studies (*p* < 0.10; *I*^2^ > 25%). Otherwise, a fixed-effect model was used.

## Results

**Fig. 1:**
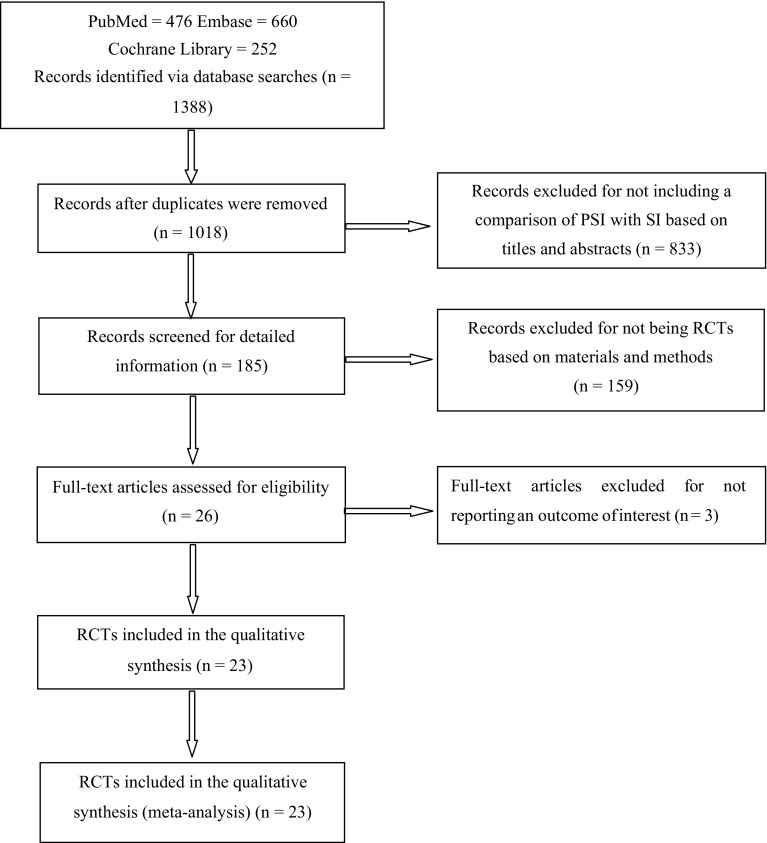
Flow diagram shows the process of selecting studies to be included in the review

The initial searches produced 1388 studies, of which 370 were duplicates and 833 were excluded because the title and abstract were irrelevant. The remaining 185 studies were retrieved for evaluation of the materials and methods, and 159 of these articles were excluded because they did not include a comparison with SI or were not RCTs; furthermore, three full-text articles were excluded because they did not report an outcome of interest. The remaining 23 RCTs [1, 4–6, 10, 13, 15, 16, 20–23, 25, 38, 39, 43, 48, 55–58, 61, 64] were included in our meta-analysis. A flow diagram detailing the study selection is shown in Fig. 1. A total of 2058 patients who underwent TKA were included in this study. Details of the study characteristics and participant demographics are shown in Tables 1 and 2. Heterogeneity, 95% CIs, and *p* values of the research parameters are shown in Table 3.

**Table 1:** Characteristics of the included studies

References	Country	Improved Jadad Rating Scale	No. of patients		Mean age (years)	
			PSI	SI	PSI	SI
Abane et al. [1]	France	5	70	70	67.8 (47–84)	70.4 (54–83)
Boonen et al. [4]	Netherlands	7	90	90	69.0 ± 8.0	65.0 ± 8.8
Boonen et al. [5]	Netherlands	7	90	90	69.0 ± 8.0	65.0 ± 8.8
Chareancholvanich et al. [6]	Thailand	5	40	40	69.5 (55–84)	70.3 (53–85)
De et al. [10]	Belgium	5	20	24	NR	NR
Gan et al. [13]	China	4	35	35	68.5 ± 4.8	67.8 ± 3.4
Hamilton et al. [15]	USA	4	26	26	68.1 (52–86)	68.1 (52–86)
Huijbregts et al. [16]	Australia	5	69	64	66.7 ± 9.1	69.0 ± 9.6
Khuangsirikul et al. [20]	Thailand	3	40	40	NR	NR
Kotela and Kotela [21]	Poland	5	49	46	66.1 ± 8.4	68.6 ± 9.9
Kotela et al. [22]	Poland	5	49	46	66.1 ± 8.4	68.6 ± 9.9
Kosse et al. [23]	Netherlands	5	21	21	62.7 ± 4.5	63.4 ± 4.2
Maus et al. [25]	Germany	5	59	66	68.1 ± 8.5	71.5 ± 8.1
Parratte et al. [38]	France	4	20	20	NR	NR
Pietsch et al. [39]	Australia	7	40	40	71.4 ± 6.6	69.2 ± 9.4
Roh et al. [43]	Korea	7	42	48	70 ± 7.2	70 ± 5.1
Silva et al. [48]	Portugal	4	23	22	73 (67–78)	74 (70.5–80)
Vundelinckx et al. [55]	Belgium	5	31	31	64.65 ± 8.23	68.19 ± 8.48
Victor et al. [56]	Belgium	5	64	64	67 (52–87)	66 (36–92)
Vide et al. [57]	Portugal	5	47	48	67.8 ± 8.4	69.3 ± 6.5
Van et al. [58]	Norway	5	44	50	67 ± 8.8	64 ± 6.9
Woolson et al. [61]	USA	5	22	26	NR	NR
Yan et al. [64]	China	5	30	30	67.5 ± 8.0	69.5 ± 8.4

*PSI* patient-specific instrumentation, *SI* standard instrumentation, *NR* not reported

**Table 2:** Characteristics of the included studies

Study	Pre-imaging	Gender (F/M)		BMI		PSI system
		PSI	SI	PSI	SI	
Abane et al. [1]	MRI	27/43	40/30	28.8 (20–40)	28.6 (20–40)	Smith & Nephew, Memphis, TN, USA
Boonen et al. [4]	MRI	56/34	50/40	30.3	29.5	Biomet, Inc., Warsaw, IN, USA
Boonen et al. [5]	MRI	56/34	50/40	30.3 (22.9–40.7)	29.5 (21.3–42.7)	Vanguard Complete Knee System
Chareancholvanich et al. [6]	MRI	34/6	36/4	27.7 (20.2–44.15)	28.0 (22–39.6)	Zimmer, Warsaw, IN, USA
De et al. [10]	MRI	NR	NR	NR	NR	Biomet, Inc., Warsaw, IN, USA
Gan et al. [13]	CT	25/10	26/9	NR	NR	Stryker, Mahwah, NJ, USA
Hamilton et al. [15]	CT	12/14	19/7	30.9 (21.5–39.6)	31.1 (22–38.4)	TruMatch, DePuy Orthopaedics, Warsaw, IN, USA
Huijbregts et al. [16]	MRI	40/29	32/32	NR	NR	Smith & Nephew
Khuangsirikul et al. [20]	CT	NR	NR	NR	NR	DePuy, Warsaw, IN, USA
Kotela and Kotela [21]	CT	33/16	33/13	30.0 ± 4.6	29.6 ± 5.6	Biomet, Inc., Warsaw, IN, USA
Kotela et al. [22]	CT	33/16	33/13	30.0 ± 4.6	29.6 ± 5.6	Biomet, Inc., Warsaw, IN, USA
Kosse et al. [23]	MRI	13/8	9/12	28.1 ± 3.3	27.8 ± 3.1	Smith & Nephew, Memphis, TN, USA
Maus et al. [25]	MRI	33/26	43/23	31.8 ± 6.1	30.6 ± 5.3	Aesculap AG, Tuttlingen
Parratte et al. [38]	MRI	NR	NR	NR	NR	Zimmer, Warsaw, IN, USA
Pietsch et al. [39]	MRI	27/13	21/19	29.0 ± 3.5	30.8 ± 4.9	Genera, Zimmer, Warsaw, IN, USA
Roh et al. [43]	CT	39/3	43/5	27 ± 4.2	27 ± 2.7	Biomet, Inc., Warsaw, IN, USA
Silva et al. [48]	MRI	NR	NR	NR	NR	Vanguard, Biomet, Inc
Vundelinckx et al. [55]	MRI	16/15	20/11	27.61 ± 3.82	31.11 ± 5.25	Smith & Nephew
Victor et al. [56]	MRI	43/21	43/21	NR	NR	Biomet, Inc., Warsaw, IN, USA; DePuy, Inc., Warsaw, IN, USA; Smith & Nephew, Inc., Memphis, TN, USA; Zimmer, Inc., Warsaw, IN, USA
Vide et al. [57]	MRI	32/15	33/15	31.0	30.3	Smith & Nephew, Inc., Memphis, TN, USA
Van et al. [58]	MRI	30/14	32/18	31 ± 4.9	29 ± 4.6	Biomet, Inc., Warsaw, IN, USA
Woolson et al. [61]	CT	NR	NR	NR	NR	NR
Yan et al. [64]	MRI	17/13	24/6	NR	NR	Zimmer, Warsaw, IN, USA

*PSI* patient-specific instrumentation, *SI* standard instrumentation, *NR* not reported

**Table 3:** Heterogeneities, 95% CIs, and *p* values of research parameters

Research parameters	Heterogeneity (*I*^2^) (%)	95% CI	*p* value
Mechanical axis of the limb	68	− 0.41 to 0.23	n.s.
Outliers of the mechanical axis of the limb	41	0.72 to 1.24	n.s.
Femoral coronal alignment	79	− 0.41 to 0.17	n.s.
Outliers of the femoral coronal alignment	37	0.57 to 1.30	n.s.
Tibial coronal alignment	62	− 0.12 to 0.30	n.s.
Outliers of the tibial coronal alignment	46	0.75 to 2.49	n.s.
Femoral sagittal alignment	83	− 1.40 to 0.41	n.s.
Outliers of the femoral sagittal alignment	46	0.84 to 1.35	n.s.
Tibial sagittal alignment	50	− 0.81 to 0.04	n.s.
Outliers of the tibial sagittal alignment	57	0.92 to 1.86	n.s.
Femoral axial alignment	48	− 0.71 to − 0.21	0.0004
Outliers of the femoral axial alignment	32	0.45 to 1.29	n.s.
Operative time	78	− 10.95 to − 3.75	< 0.0001
Perioperative blood loss	74	− 146.65 to − 20.18	0.01
Length of hospital stay	19	− 0.40 to 0.07	n.s.

*n.s*. non-significant

**Fig. 2:**
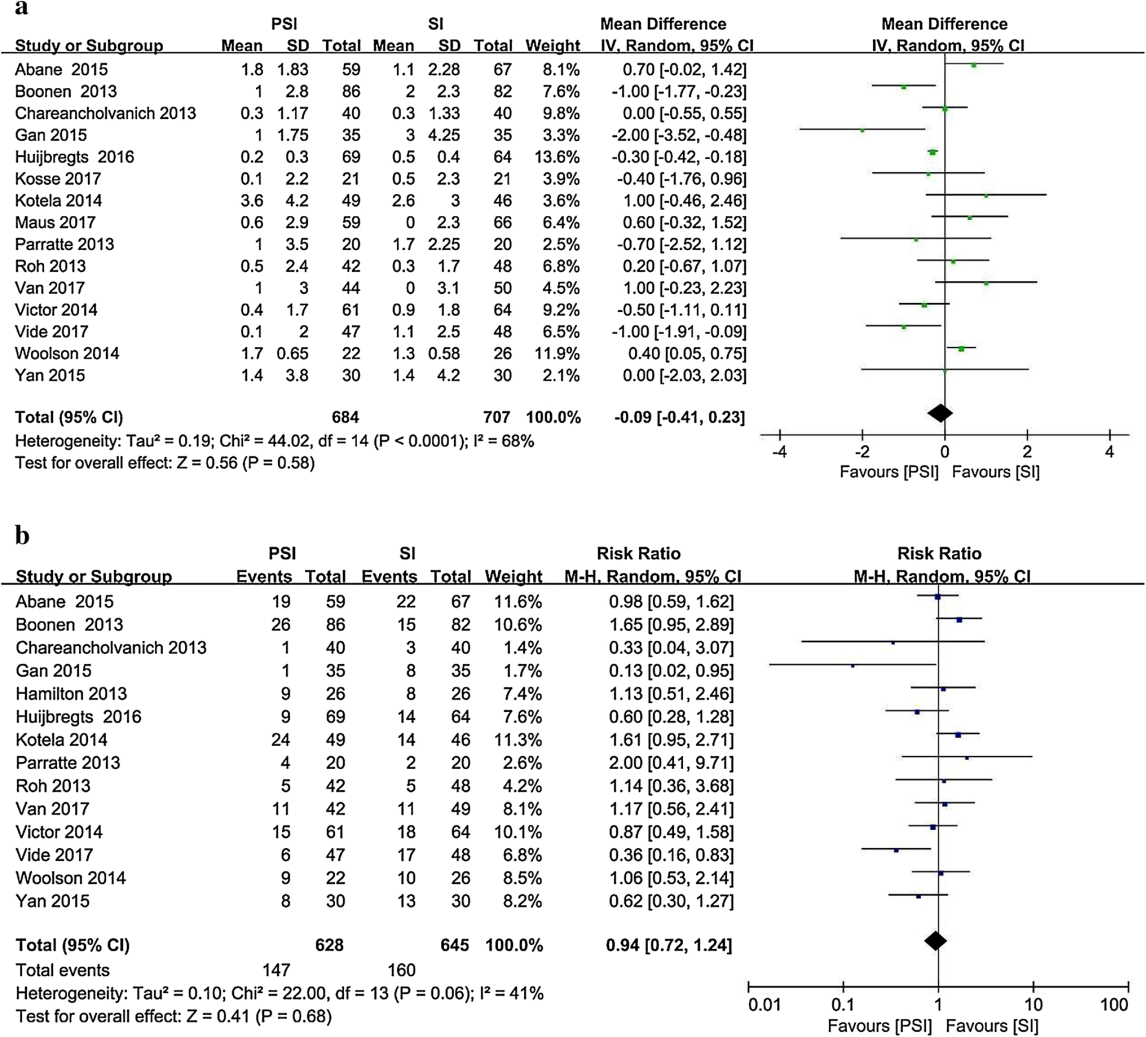
Postoperative HKA angle in the PSI and SI groups: **a** absolute deviation from the target alignment (180°) and **b** number of outliers (> 3° from the target alignment)

Fifteen studies [1, 4, 6, 13, 16, 21, 23, 25, 38, 43, 56–58, 61, 64] reported the postoperative mechanical axis of the limb (expressed as the hip–knee–ankle angle, HKA) as the mean and standard deviation (Fig. 2). Fourteen studies [1, 4, 6, 13, 15, 16, 21, 38, 43, 56–58, 61, 64] involving 1391 patients and reporting postoperative outliers of the mechanical axis of the limb were included. The PSI group contained 147 outliers among 628 patients, whereas 160 outliers were recorded among the 645 patients in the SI group (23.4% vs. 24.8%).

**Fig. 3:**
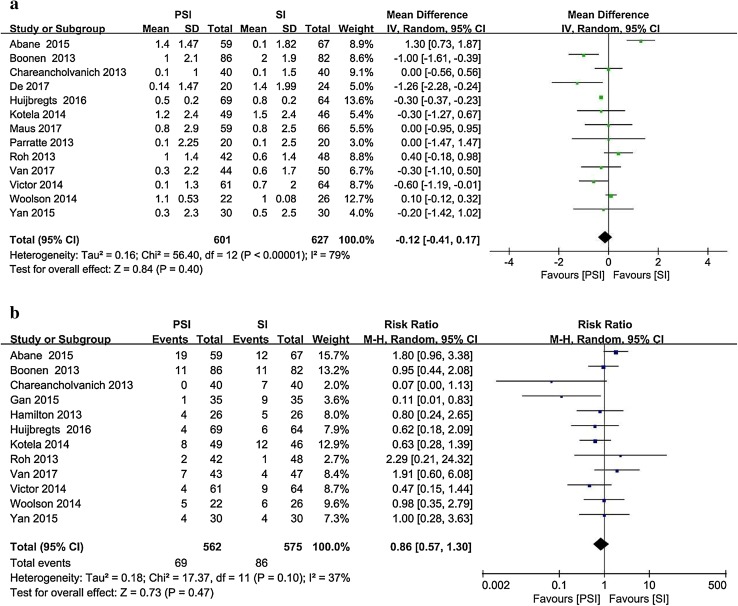
Postoperative femoral coronal alignment in the PSI and SI groups: **a** absolute deviation from the target alignment (90°) and **b** number of outliers (> 3° from the target alignment)

Thirteen studies [1, 4, 6, 9, 16, 21, 25, 38, 43, 56, 58, 61, 64] reported the postoperative femoral coronal alignment as the mean and standard deviation (Fig. 3). The target alignment was 90°. Twelve studies [1, 4, 6, 13, 14, 16, 21, 43, 56, 58, 61, 64] involving 1137 patients and reporting postoperative outliers of the femoral coronal alignment were included. The PSI group contained 69 outliers among 562 patients, whereas 86 outliers were recorded among the 575 patients in the SI group (12.3% vs. 15.0%).

**Fig. 4:**
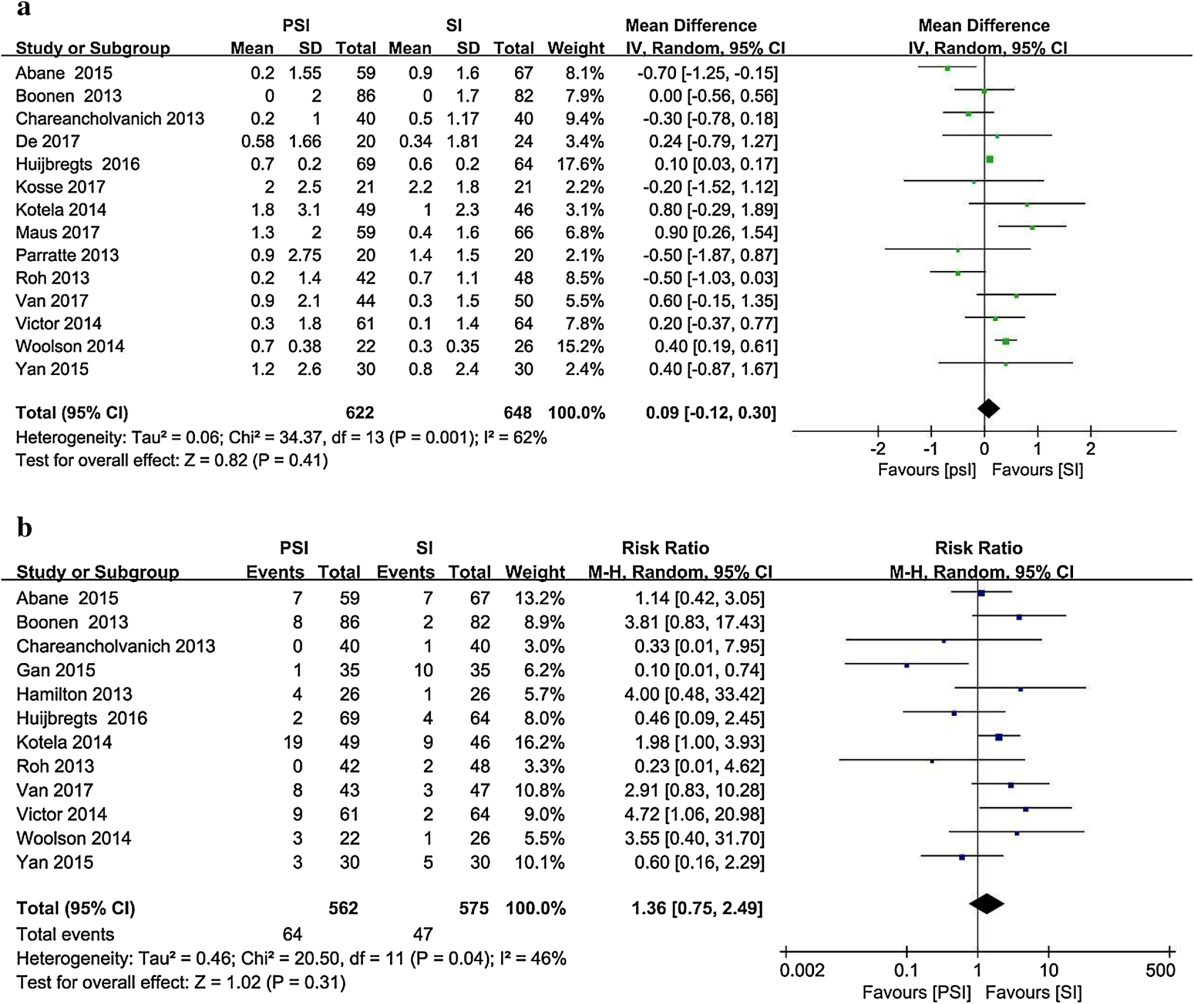
Postoperative tibial coronal alignment in the PSI and SI groups: **a** absolute deviation from the target alignment (90°) and **b** number of outliers (> 3° from the target alignment)

Fourteen studies [1, 4, 6, 9, 16, 21, 23, 25, 38, 43, 56, 58, 61, 64] reported the postoperative tibial coronal alignment as the mean and standard deviation (Fig. 4). Twelve studies [1, 4, 6, 13, 15, 16, 21, 43, 56, 58, 61, 64] involving 1137 patients and reporting postoperative outliers of the tibial coronal alignment were included. The PSI group contained 64 outliers among 562 patients, whereas 47 outliers were recorded among the 575 patients in the SI group (11.4% vs. 8.2%).

**Fig. 5:**
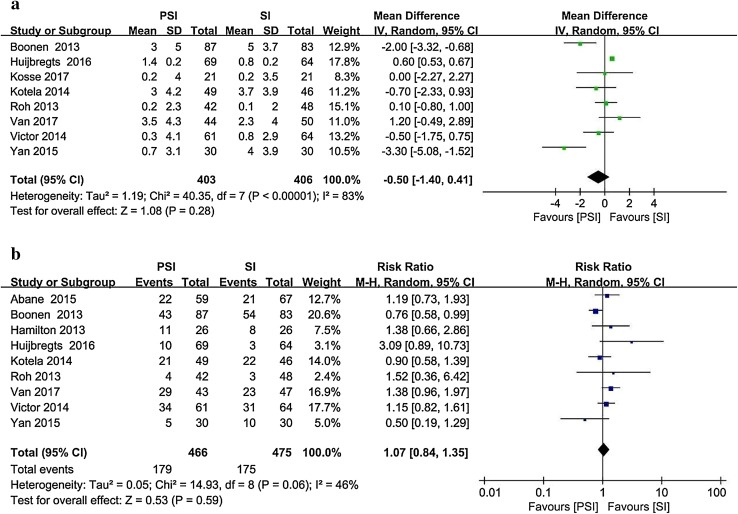
Postoperative femoral sagittal alignment in the PSI and SI groups: **a** absolute deviation from the target alignment and **b** number of outliers (> 3° from the target alignment)

Eight studies [4, 16, 21, 23, 43, 56, 58, 64] reported the postoperative femoral sagittal alignment as the mean and standard deviation (Fig. 5). The target alignment was defined differently in the literature. The absolute deviation between the actual measured value and the target alignment was recorded. Nine studies [1, 4, 15, 16, 21, 43, 56, 58, 64] involving 941 patients that reported postoperative outliers of the femoral sagittal alignment were included. The PSI group contained 179 outliers among 466 patients, whereas 175 outliers were recorded among the 475 patients in the SI group (38.4% vs. 36.8%).

**Fig. 6:**
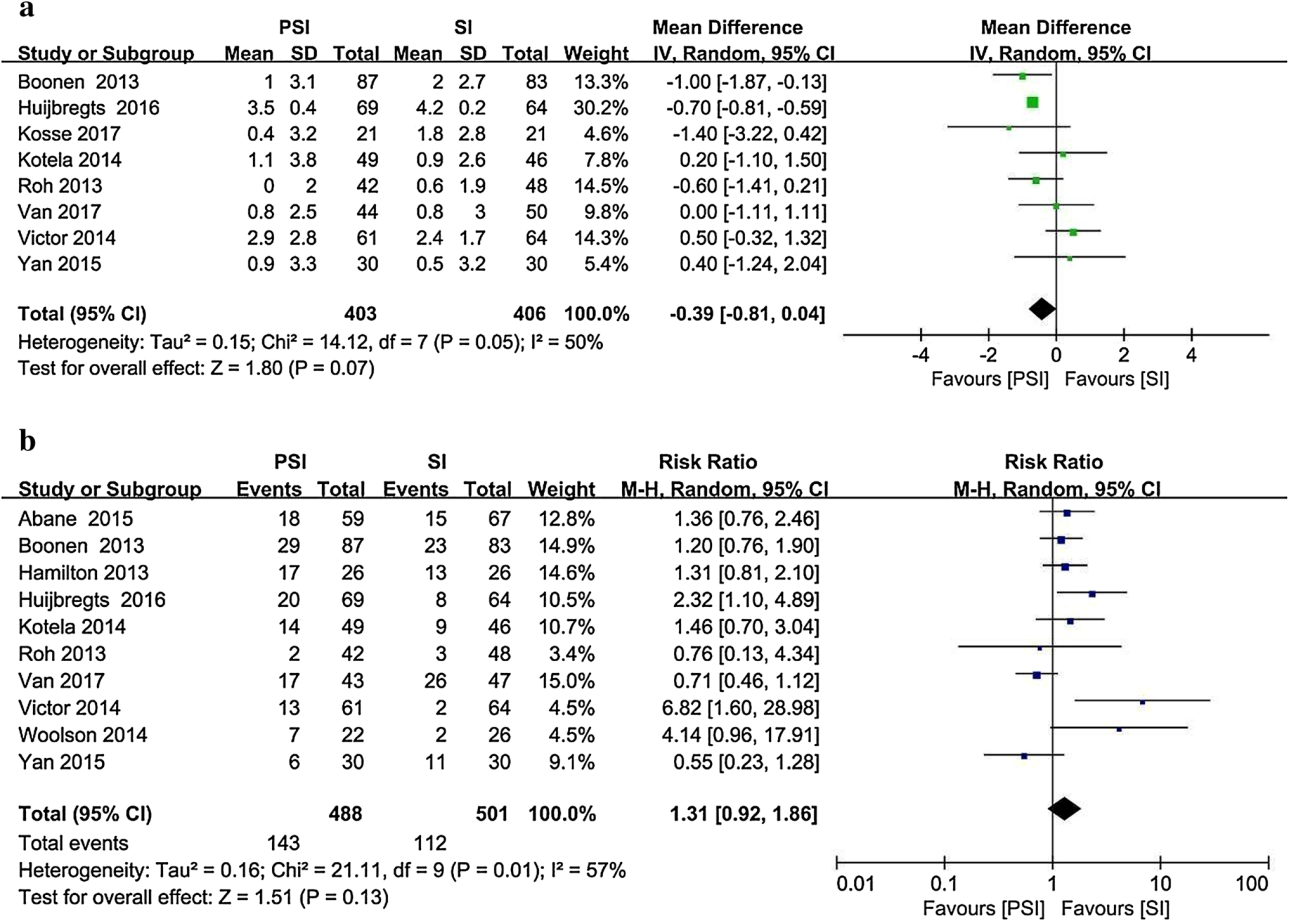
Postoperative tibial sagittal alignment in the PSI and SI groups: **a** absolute deviation from the target alignment and **b** number of outliers (> 3° from the target alignment)

Eight studies [4, 16, 21, 23, 43, 56, 58, 64] reported the postoperative tibial sagittal alignment as the mean and standard deviation (Fig. 6). The target alignment was defined differently in the literature. The absolute deviation between the actual measured value and the target alignment was recorded. Ten studies [1, 4, 15, 16, 21, 43, 56, 58, 61, 64] involving 989 patients and reporting the postoperative outliers of the tibial sagittal alignment were included. The PSI group included 143 outliers among 488 patients, whereas 112 outliers were recorded among the 501 patients in the SI group (29.3% vs. 22.4%).

**Fig. 7:**
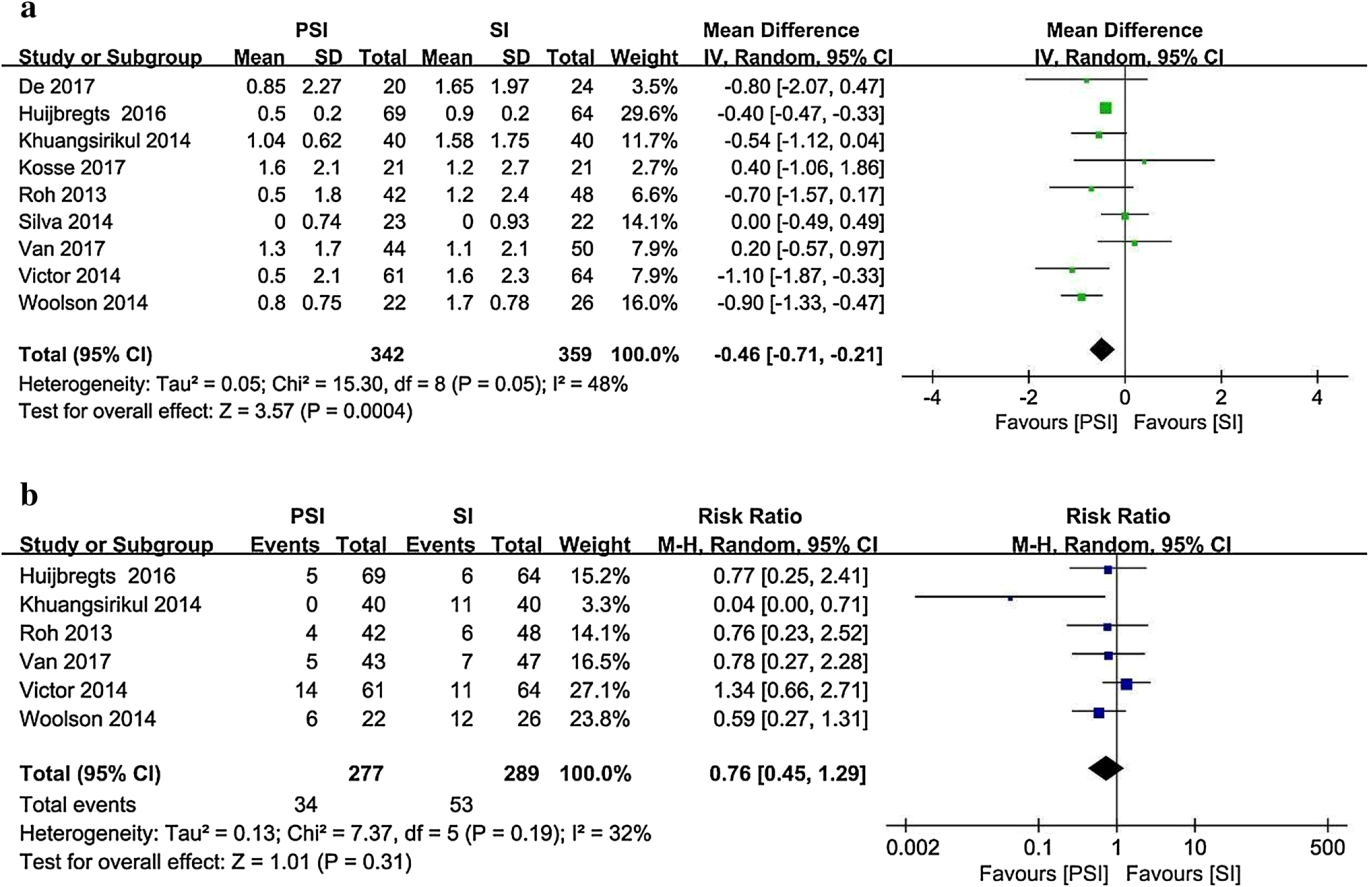
Postoperative femoral axial alignment in the PSI and SI groups: **a** absolute deviation from the target alignment and **b** number of outliers (> 3° from the target alignment)

Nine studies [4, 9, 16, 20, 43, 48, 56, 58, 61] reported the postoperative femoral axial alignment as the mean and standard deviation (Fig. 7). The target alignment was parallel to the transcondylar line. Six studies [16, 20, 44, 56, 58, 61] involving 566 patients and reporting postoperative outliers of the femoral axial alignment were included. The PSI group contained 34 outliers among 277patients, whereas 53 outliers were recorded among the 289 patients in the SI group (12.3% vs. 18.3%).

**Fig. 8:**
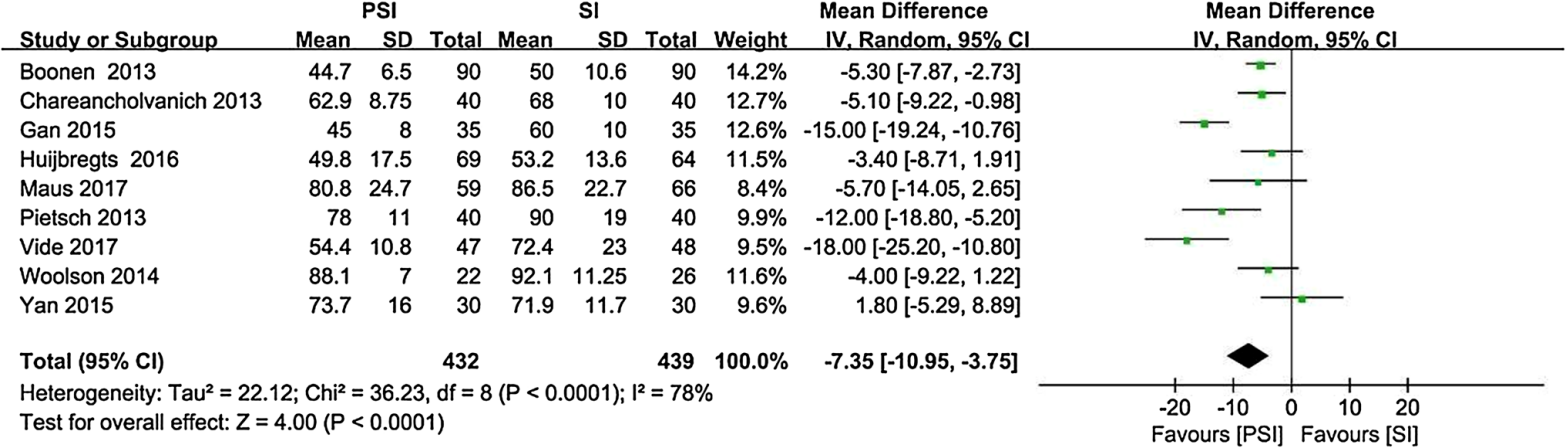
Operative time with PSI versus SI

**Fig. 9:**
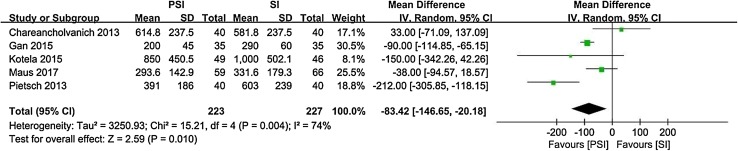
Perioperative blood loss with PSI versus SI

**Fig. 10:**
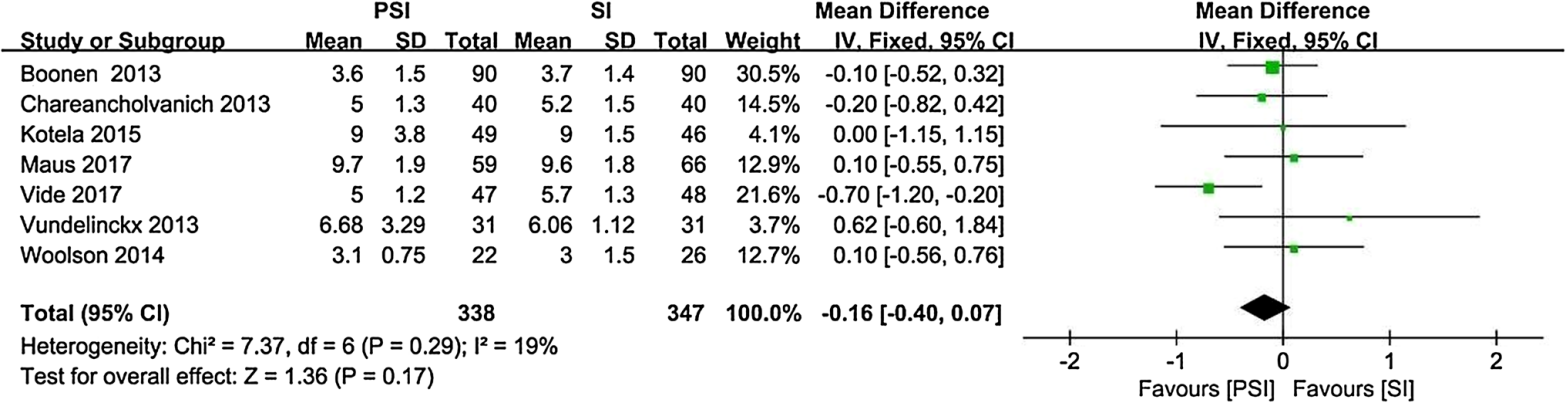
Length of hospital stay with PSI versus SI

Nine studies [4, 6, 13, 16, 25, 39, 57, 61, 64] reported the operative time as the mean and standard deviation (Fig. 8). Five studies [6, 13, 22, 25, 39] reported the perioperative blood loss as the mean and standard deviation (Fig. 9). Seven studies [4, 6, 22, 25, 55, 57, 61] reported the length of hospital stay as the mean and standard deviation (Fig. 10).

## Discussion

The most important findings of the present study were that PSI resulted in approximately 0.4° less deviation from the transcondylar line, reduced perioperative blood loss by 90 ml and reduced the operative time by an average of 7 min compared to SI. No significant differences between PSI and SI were found with respect to alignment of the remaining components, number of outliers, and length of hospital stay.

The effectiveness of PSI compared to that of SI is not completely clear, and the existing data are conflicting. The present study produced results that are consistent with some published studies showing that PSI and SI exhibited no significant difference in mechanical alignment [12, 17, 26, 27, 31, 40, 41, 49, 65]. However, other published studies reached a conclusion opposite to that of the present investigation [3, 44, 59]. Postoperative mechanical alignment is critical to the long-term survival of the prosthesis. Therefore, more well-designed, high-quality, long-term RCTs are needed monitor the survival of the prosthesis. A few studies showed a significant reduction in outliers of the mechanical alignment for PSI compared to SI [3, 11, 28, 33, 44]. However, in the present study, no evident difference in outliers of mechanical alignment was found between PSI and SI. The existing studies showed no significant difference in the coronal and sagittal alignment of the femoral component [12, 17, 26, 40, 65]. Several studies showed no significant difference in the coronal and sagittal alignment of the tibial component [17, 26, 40]. In fact, the mechanical alignment was ultimately determined by the coronal alignment of the femoral and tibial components. Therefore, it was reasonable that we concluded that PSI and SI produced no evident difference in the mechanical alignment. PSI and SI had no evident difference in outliers of the coronal and sagittal alignment of the femoral and tibial component. Two published papers showed the same outcome [26, 65].

PSI showed approximately 0.4° less deviation from the transcondylar line than SI. Theoretically, the femoral axial alignment should be parallel to the transcondylar line. The clinical relevance of a 0.4° deviation is questionable despite the statistically significant difference. In the future, additional clinically relevant studies of femoral axial alignment should be conducted.

PSI reduced the operative time by an average of 7 min compared to SI. Several published studies supported our opinions [29, 32, 44, 50] due to simplification of the operative procedures. However, the clinical relevance of a 7-min reduction is questionable, despite the statistically significant difference. Additional studies should be conducted regarding the clinical relevance of a reduction in operative time in the future. PSI could reduce the perioperative blood loss by approximately 90 ml compared to SI because PSI avoids invasion of the femoral medullary cavity and shortens the operative time. Published studies have reported analogous outcomes [24, 34, 47].

There are some limitations to this study. First, the data showed large heterogeneity among the included studies, which may have affected the analysis of the results. Second, some of the data conversions in the articles may have affected the analysis of the results.

## Conclusion

PSI has advantages for axial alignment of the femoral component, operative time, and perioperative blood loss compared to SI. However, no significant differences were observed between PSI and SI with respect to the alignment of the remaining components, number of outliers, and length of hospital stay. High-quality, long-term RCTs are needed to determine whether PSI is superior to SI in other respects.
